# Combined assessment of body mass index and triglyceride-glucose index reveals metabolic risk phenotypes associated with semen quality impairment

**DOI:** 10.3389/fendo.2026.1960020

**Published:** 2026-09-24

**Authors:** Yannan Zhao, Yue Zhao, Yan Zhao, Li Jiang, Shuaishuai Guo, Lihong Jia

**Affiliations:** 1 Shenyang Clinical Medical Research Center for Assisted Reproduction, Shenyang Women’s and Children’s Hospital, Shenyang, China; 2 Liaoning Key Laboratory of Assisted Reproduction, Shenyang, China; 3 Key Laboratory of Environmental Stress and Chronic Disease Control & Prevention, Ministry of Education, China Medical University, Shenyang, China; 4 Department of Child and Adolescent Health, School of Public Health, China Medical University, Shenyang North New Area, Shenyang, China

**Keywords:** body mass index, male reproductive health, metabolic risk phenotype, semen quality, triglyceride-glucose index

## Abstract

**Background:**

Body mass index (BMI) has been associated with impaired semen quality, but evidence remains inconsistent, partly because BMI does not adequately reflect metabolic health. The triglyceride-glucose (TyG) index, a surrogate for insulin resistance (IR), is increasingly recognized as a potential contributor to impaired semen quality. However, whether BMI and TyG are jointly associated with semen quality has not been evaluated.

**Methods:**

This retrospective cross-sectional study included 804 men from a fertility clinic. Associations of BMI and TyG with semen parameters were evaluated using multivariable linear and logistic regression models. Receiver operating characteristic, net reclassification improvement (NRI), and integrated discrimination improvement analyses were performed to assess model discrimination and reclassification. Exploratory mediation analyses estimated the extent to which TyG accounted for the observed associations between BMI and semen quality.

**Results:**

Elevated BMI and TyG were independently associated with lower total motility, progressive motility, and normal morphology. Men with both high BMI and high TyG showed the highest odds of reduced total motility (OR 3.63, 95% CI:1.99, 6.61), progressive motility (OR 3.21, 95% CI:1.74, 5.90), and normal forms (OR 2.78, 95% CI:1.57, 4.92). Men with low BMI but high TyG showed numerically higher impairment rates than men with high BMI but low TyG across motility and morphology outcomes. The incremental NRI of TyG beyond BMI ranged from 0.212 to 0.321 for these parameters (all P < 0.01). Exploratory mediation analyses indicated that TyG statistically accounted for 35.9%, 33.9%, and 28.5% of the observed association between BMI and total motility, progressive motility, and normal forms, respectively.

**Conclusions:**

BMI and TyG index were independently associated with impaired sperm motility and normal morphology, while the combination of BMI and TyG provided additional risk stratification. Elevated TyG may help identify metabolically at-risk men who are not fully captured by BMI alone, although further validation is required.

## Introduction

Infertility affects roughly one in six people worldwide, and male factors contribute to nearly 50% of infertility cases (1, 2). Pooled data spanning several decades reveal deterioration in mean sperm quality among men from North America and Europe, and this downward trajectory has been confirmed in Asian populations as well (3, 4). The causes of reduced semen quality are complex and multifaceted; metabolic disturbances are gaining recognition as substantial contributors (5).

Among various metabolic factors, body mass index (BMI) has been the most frequently studied exposure in male fertility research. The biological plausibility linking BMI to male fertility involves disrupted hypothalamic-pituitary-gonadal (HPG) axis signaling, endocrine disruptors, and epigenetic changes (6, 7). Epidemiological evidence, however, remains inconsistent. Some reports suggest that obesity correlates with decreased semen volume, total count, and progressive motility (8, 9). Others have found no such associations (10, 11). This inconsistency may partly reflect an inherent limitation of BMI: it quantifies body mass but not the complexity of metabolic health. In most fertility settings, BMI remains the primary metabolic indicator captured, while indices of insulin resistance (IR), such as triglyceride-glucose (TyG) index, are rarely incorporated into routine assessment.

IR, often co-occurring with obesity, has recently been highlighted as a risk factor for abnormal semen quality parameters (12, 13). The TyG index, calculated from triglycerides and glucose, shows good agreement with conventional IR assessments (14). The index has demonstrated predictive value for metabolic disorders including cardiovascular disease (15) and type 2 diabetes (16). However, its contribution to male reproductive health has received limited attention. A recent cross-sectional study linked higher TyG levels to increased likelihood of oligozoospermia, azoospermia, and elevated sperm DNA fragmentation (17). Evidence concerning TyG and conventional semen parameters remains limited, and most existing studies have evaluated TyG in isolation.

Obesity and IR frequently coexist and share interconnected metabolic pathways (18, 19). Individuals with normal BMI may still exhibit IR, whereas obese individuals are not uniformly metabolically impaired. Relying BMI alone may misclassify metabolic risk and fail to identify individuals at increased reproductive risk in fertility assessment. However, no previous study has evaluated semen quality in relation to the combination of BMI and TyG. We therefore examined the independent associations of BMI and TyG with semen quality impairment and evaluated risk patterns across combined BMI and TyG metabolic phenotypes. We also performed exploratory mediation analysis to quantify the extent to which TyG accounted for part of the observed association between BMI and semen quality.

## Methods

### Study population

This retrospective cross-sectional study included men aged 20 years or older who underwent fertility evaluation at the Reproductive Center of Shenyang Women and Children’s Hospital, Shenyang, China, between June 2023 and March 2024. The exclusion criteria were as follows: (1) moderate or severe varicocele, hydrocele, any genital malformation, history of genital surgery, or trauma; (2) inguinal hernia, epididymo-orchitis, reproductive system infection, mumps, or testicular tumor; (3) vas deferens obstruction or absence, or azoospermia; (4) genetic factors (chromosomal abnormalities or AZF microdeletion). After excluding individuals with underweight (n=9) and incomplete data (n=6), we included 804 men in the final analysis. The study was approved by the Ethics Committee of Shenyang Women’s and Children’s Hospital (PJ-SC-2026-030).

### Semen analysis

We performed semen assessments in compliance with the procedures specified in the 2010 WHO laboratory manual, which was the routine laboratory standard used at our center during the study period. For each participant, semen parameters were assessed using a single semen sample collected by masturbation after 2–7 days of sexual abstinence. Samples were allowed to liquefy at 37 °C for 30 minutes. Sperm concentration and motility were assessed using a computer-assisted semen analysis (CASA) system. Sperm motility was categorized as total motility (progressive plus non-progressive motility) and progressive motility. Measurements were repeated when discrepancies exceeded predefined quality-control limits. We conducted sperm morphology using the Diff-Quik^®^ stain. Total sperm number was calculated as semen volume multiplied by sperm concentration.

### Data collection and related definitions

Demographic and clinical information, including age, education level, smoking status, and laboratory measurements (fasting blood glucose (FBG), triglycerides (TG), total cholesterol (TC), high-density lipoprotein cholesterol (HDL-C), and low-density lipoprotein cholesterol (LDL-C)) were extracted from electronic medical records. Diabetes and dyslipidemia were defined according to standard diagnostic criteria.

### Calculation formula

The TyG index was calculated via the formula: TyG=ln [TG (mg/dL) × FBG (mg/dL)/2]. BMI was calculated as weight (kg) divided by the square of height (m^2^). According to the World Health Organization (WHO) classification (20), BMI was categorized as Low-BMI: normal weight (18.5 ≤ BMI < 25.0); Middle-BMI: overweight (25.0 ≤ BMI < 30.0); and High-BMI: obesity (BMI ≥ 30.0).

### Statistical analyses

All continuous variables exhibited non-normal distributions and were presented as median (interquartile range, IQR). Categorical variables were expressed as frequency (percentage). Comparisons of baseline characteristics across BMI and TyG categories were performed using the Kruskal-Wallis H test for continuous variables and the chi-square (χ²) test or Fisher’s exact test for categorical variables, as appropriate. All sperm quality parameters were natural log-transformed (ln) prior to regression analysis to better approximate a normal distribution. No universally accepted clinical cut-off for TyG exists in reproductive populations. The TyG index was dichotomized at the sample median (8.94) and cross-classified with BMI categories to define six combined BMI–TyG phenotypes: L-BMI/L-TyG (reference), M-BMI/L-TyG, H-BMI/L-TyG, L-BMI/H-TyG, M-BMI/H-TyG, and H-BMI/H-TyG.

We constructed multivariable linear regression models to evaluate the independent and joint associations of BMI and TyG index with sperm quality parameters. Regression coefficients (β) from ln-transformed outcomes were back-transformed to percentage changes using (e^β − 1) × 100, which expresses the relative difference in semen parameters associated with exposure categories. We also modeled TyG as a continuous variable (per 1-SD increase) to assess the robustness of the findings. Two models were constructed: in model 1, age was adjusted; in model 2, age, education level, abstinence duration, smoking status and dyslipidemia were adjusted. To further analyze the association between BMI, TyG, and their combined categories and the risk of low semen quality, we used logistic regression models to estimate odds ratios (ORs) and 95% confidence intervals (CIs). Semen decline was defined dichotomously for each parameter using the WHO 2010 lower reference limits as cutoffs: semen volume < 1.5 mL, sperm concentration < 15 × 10^6^/mL, total sperm count < 39 × 10^6^ per ejaculate, total motility < 40%, progressive motility < 32%, and normal forms < 4% (21). Models were adjusted for age, education level, abstinence duration, smoking status and dyslipidemia.

Multiplicative interaction between BMI and TyG categories was assessed by including BMI category and TyG category product term in the linear regression models. Interaction estimates were expressed as percentage changes with 95% CIs after back-transformation from ln-transformed semen parameters.

Receiver operating characteristic (ROC) curve analysis was performed to evaluate whether adding BMI and/or the TyG index to a basic clinical model improved model discrimination for dichotomized semen quality outcomes. We calculated the area under the ROC curve (AUC) for each model. Furthermore, continuous net reclassification improvement (NRI) and integrated discrimination improvement (IDI) index were calculated to assess incremental reclassification. The basic model included age, education level, abstinence duration, smoking status and dyslipidemia.

Exploratory mediation analyses were performed to assess whether TyG accounted for part of the observed associations between BMI and semen parameters. We defined exposure as BMI, the mediator as the TyG, and outcomes as semen parameters. Indirect, direct, and total effects, along with the indirect effect proportion, were estimated after covariate adjustment (same as above).

We also evaluated potential effect modification by age (<35 vs. ≥35 years). Interaction terms between age group and combined BMI and TyG categories were included in the fully adjusted models. All statistical analyses were performed using R software version 4.3.2 (R Foundation for Statistical Computing, Vienna, Austria). Two-sided P < 0.05 was considered statistically significant.

## Results

### Baseline characteristics

A total of 804 men were included in the analysis. The median age was 34.0 years (IQR, 31.0-37.0 years), while the median BMI and TyG index were 26.1 kg/m² (IQR, 23.7-29.0) and 8.9 (IQR, 8.5-9.4), respectively (**Table 1**). The overall distribution of semen quality parameters is presented in **Supplementary Table 1**. Across both BMI and TyG strata, significant differences were observed in education level, FBG, TG, TC, HDL-C, LDL-C, dyslipidemia, and diabetes (all P < 0.01). Increasing BMI and TyG levels were accompanied by progressively less favorable metabolic profiles. Total motility, progressive motility, and normal forms were significantly lower across increasing BMI categories and in men with higher TyG levels (all P ≤ 0.001), whereas semen volume, sperm concentration, and total sperm number did not differ significantly between groups.

**Table 1:** Baseline characteristics of participants by BMI and TyG index categories.

Characteristic	Overall (n=804)	BMI <25.0 (n=311)	BMI 25.0-29.9 (n=348)	BMI ≥30.0 (n=145)	P value (BMI)	TyG <8.94 (n=403)	TyG ≥8.94 (n=401)	P value (TyG)
Age, median (IQR), years	34.0 (31.0, 37.0)	34.0 (32.0, 36.0)	34.0 (32.0, 37.0)	34.0 (30.0, 36.0)		34.0 (32.0, 37.0)	34.0 (31.0, 37.0)	
<35, n (%)	445 (55.3)	181 (58.2)	179 (51.4)	85 (58.6)	0.151	223 (55.3)	222 (55.4)	1.000
≥35, n (%)	359 (44.7)	130 (41.8)	169 (48.6)	60 (41.4)		180 (44.7)	179 (44.6)	
Race, Han, n (%)	777 (96.6)	301 (96.8)	337 (96.8)	138 (95.2)	0.649	386 (95.8)	390 (97.3)	0.336
Education, n (%)					0.017			0.006
High school and lower	236 (29.4)	94 (30.2)	101 (29.0)	41 (28.3)		112 (27.8)	124 (30.9)	
Junior college	174 (21.6)	68 (21.9)	88 (25.3)	18 (12.4)		106 (26.3)	68 (17.0)	
College or above	394 (49.0)	149 (48.0)	159 (45.7)	86 (59.3)		185 (45.9)	209 (52.1)	
Current smoking, n (%)	324 (40.3)	118 (37.9)	138 (39.7)	68 (46.9)	0.181	158 (39.2)	166 (41.4)	0.565
Current drinking, n (%)	82 (10.2)	34 (10.9)	32 (9.2)	16 (11.0)	0.735	45 (11.2)	37 (9.2)	0.415
abstinence duration (days)					0.550			0.609
≤3	181 (22.5)	74 (23.8)	82 (23.6)	25 (17.2)		85 (21.1)	96 (23.9)	
4-5	363 (45.1)	141 (45.3)	153 (44.0)	69 (47.6)		187 (46.4)	176 (43.9)	
>5	260 (32.3)	96 (30.9)	113 (32.5)	51 (35.2)		131 (32.5)	129 (32.2)	
BMI, median (IQR), kg/m²	26.1 (23.7, 29.0)	22.9 (21.2, 23.9)	27.4 (26.1, 28.4)	31.6 (30.7, 33.1)	<0.001	25.0 (22.6, 27.6)	27.7 (24.8, 30.1)	<0.001
TyG index, median (IQR)	8.9 (8.5, 9.4)	8.6 (8.3, 9.1)	9.0 (8.6, 9.5)	9.3 (8.9, 9.7)	<0.001	8.5 (8.2, 8.7)	9.4 (9.1, 9.8)	<0.001
FBG, median (IQR), mmol/L	5.7 (5.4, 6.1)	5.7 (5.4, 6.0)	5.8 (5.4, 6.1)	5.9 (5.6, 6.4)	<0.001	5.6 (5.4, 5.9)	5.8 (5.6, 6.4)	<0.001
TG, median (IQR), mmol/L	1.7 (1.1, 2.6)	1.3 (0.9, 1.9)	1.7 (1.2, 2.9)	2.2 (1.6, 3.1)	<0.001	1.1 (0.8, 1.4)	2.6 (2.0, 3.6)	<0.001
TC, median (IQR), mmol/L	5.1 (4.5, 5.8)	5.0 (4.3, 5.6)	5.1 (4.6, 5.8)	5.3 (4.8, 5.9)	0.008	4.9 (4.3, 5.5)	5.4 (4.8, 6.0)	<0.001
HDL-C, median (IQR), mmol/L	1.2 (1.1, 1.4)	1.3 (1.2, 1.5)	1.2 (1.0, 1.4)	1.1 (1.0, 1.3)	<0.001	1.3 (1.2, 1.5)	1.1 (1.0, 1.3)	<0.001
LDL-C, median (IQR), mmol/L	3.4 (2.8, 4.0)	3.2 (2.6, 3.8)	3.4 (2.9, 4.1)	3.7 (3.1, 4.2)	<0.001	3.2 (2.8, 3.8)	3.6 (3.1, 4.2)	<0.001
Dyslipidemia, n (%)	389 (48.4)	102 (32.8)	190 (54.6)	97 (66.9)	<0.001	81 (20.1)	308 (76.8)	<0.001
Diabetes, n (%)	44 (5.5)	7 (2.3)	22 (6.3)	15 (10.3)	0.001	6 (1.5)	38 (9.5)	<0.001
Semen volume, median (IQR), mL	2.5 (2.0, 3.2)	2.5 (2.1, 3.2)	2.5 (2.0, 3.2)	2.5 (2.0, 3.2)	0.522	2.5 (2.0, 3.2)	2.5 (2.0, 3.2)	0.268
Sperm concentration, median (IQR), 10^6^/mL	29.5 (15.8, 50.2)	31.6 (16.0, 50.4)	29.3 (15.9, 52.2)	26.8 (16.6, 44.1)	0.176	31.0 (16.4, 50.4)	28.5 (15.9, 47.9)	0.546
Total sperm number, median (IQR), 10^6^	72.1 (39.6, 132.4)	75.8 (39.8, 135.7)	69.0 (37.5, 132.4)	66.7 (34.9, 126.5)	0.372	69.9 (40.0, 141.9)	72.8 (37.5, 122.4)	0.336
Total motility, median (IQR), %	46.0 (34.0, 60.0)	50.0 (38.0, 65.0)	46.0 (34.0, 59.0)	42.0 (28.5, 54.5)	<0.001	50.0 (41.0, 64.0)	42.0 (29.0, 56.0)	<0.001
Progressive motility, median (IQR), %	41.0 (32.0, 54.0)	45.0 (32.0, 59.0)	41.0 (29.0, 54.0)	37.0 (23.5, 49.0)	<0.001	46.0 (34.0, 59.0)	37.0 (25.5, 51.0)	<0.001
Normal forms, median (IQR), %	4.0 (3.0, 5.0)	4.0 (3.0, 5.0)	4.0 (3.0, 5.0)	4.0 (3.0, 5.0)	0.001	4.0 (3.0, 6.0)	4.0 (2.0, 5.0)	<0.001

BMI, body mass index; TyG, triglyceride-glucose index; FBG, fasting blood glucose; TG, triglycerides; TC, total cholesterol; HDL-C, high-density lipoprotein cholesterol; LDL-C, low-density lipoprotein cholesterol. P values: Kruskal-Wallis or chi-squared test.

### Independent associations and combined associations of BMI and TyG index categories in relation to semen quality

In multivariable linear regression analyses, higher BMI was associated with lower total motility (−14.5%; 95% CI: −23.5%, −4.4%), progressive motility (−16.0%; 95% CI: −26.1%, −4.5%), and normal forms (−15.7%; 95% CI: −24.8%, −5.5%). Elevated TyG showed numerically larger reductions in these parameters, with decreases of 17.9% (95% CI: -25.0%, -10.0%), 19.4% (95% CI: -27.4%, -10.4%), and 20.2% (95% CI: -27.2%, -12.3%), respectively, and similar patterns were observed for TyG as a continuous measure (**Table 2**). Logistic regression analyses were consistent: higher BMI and TyG were associated with elevated odds of low total motility, progressive motility and normal forms, after full covariate adjustment (**Figure 1**).

**Table 2:** Estimated Percent Changes and 95% CIs for semen quality parameters in relation to independent and combined associations of BMI and TyG index categories

Variable	Semen volume, mL		Sperm concentration, 10^6^/mL		Total sperm number, 10^6^		Total motility, %		Progressive motility, %		Normal forms, %	
	Model 1	Model 2	Model 1	Model 2	Model 1	Model 2	Model 1	Model 2	Model 1	Model 2	Model 1	Model 2
BMI category												
18.5 ≤ BMI < 25 (Low)	Ref	Ref	Ref	Ref	Ref	Ref	Ref	Ref	Ref	Ref	Ref	Ref
25 ≤ BMI < 30 (Middle)	-5.1 (-11.4, 1.7)	-4.5 (-11.0, 2.4)	-2.7 (-15.3, 11.9)	-3.4 (-16.2, 11.3)	-7.6 (-20.5, 7.5)	-7.8 (-21.0, 7.6)	-10.2 (-17.5, -2.3)	-8.0 (-15.5, 0.2)	-12.1 (-20.3, -3.2)	-9.5 (-18.0, -0.2)	-11.0 (-18.2, -3.0)	-9.2 (-16.7, -0.9)
BMI ≥ 30 (High)	-3.9 (-12.0, 5.0)	-2.9 (-11.4, 6.6)	-5.5 (-20.9, 13.1)	-5.4 (-21.4, 14.0)	-9.1 (-25.1, 10.4)	-8.0 (-24.8, 12.5)	-20.2 (-28.4, -11.0)	-14.5 (-23.5, -4.4)	-22.6 (-31.7, -12.3)	-16.0 (-26.1, -4.5)	-18.2 (-26.7, -8.7)	-15.7 (-24.8, -5.5)
TyG index category												
TyG < 8.94 (Low)	Ref	Ref	Ref	Ref	Ref	Ref	Ref	Ref	Ref	Ref	Ref	Ref
TyG < 8.94 (High)	-3.3 (-9.3, 2.8)	-2.9 (-10.0, 4.9)	-3.6 (-15.0, 9.3)	-7.6 (-20.8, 7.7)	-6.9 (-18.7, 6.7)	-10.2 (-24.0, 6.1)	-21.7 (-27.4, -15.6)	-17.9 (-25.0, -10.0)	-24.3 (-30.6, -17.5)	-19.4 (-27.4, -10.4)	-19.6 (-25.5, -13.2)	-20.2 (-27.2, -12.3)
**TyG Per 1 SD**	-2.3 (-5.3, 0.8)	-2.5 (-6.3, 1.4)	0.2 (-5.9, 6.7)	-0.4 (-8.1, 8.0)	-2.1 (-8.5, 4.8)	-2.9 (-11.0, 6.0)	-15.0 (-18.1, -11.8)	-16.4 (-20.2, -12.5)	-17.1 (-20.5, -13.4)	-18.4 (-22.7, -13.9)	-13.3 (-16.6, -10.0)	-16.2 (-20.2, -12.1)
Combined variables												
L-BMI/L-TyG	Ref	Ref	Ref	Ref	Ref	Ref	Ref	Ref	Ref	Ref	Ref	Ref
M-BMI/L-TyG	-2.2 (-11.0, 7.5)	-1.8 (-10.7, 7.9)	-3.5 (-20.2, 16.5)	-4.1 (-20.7, 16.0)	-5.5 (-23.0, 16.0)	-5.7 (-23.3, 15.8)	-8.9 (-18.6, 2.0)	-9.2 (-18.9, 1.5)	-11.6 (-22.4, 0.7)	-12.0 (-22.7, 0.2)	-8.5 (-18.4, 2.6)	-8.3 (-18.3, 2.8)
H-BMI/L-TyG	-5.8 (-18.9, 9.3)	-4.9 (-18.1, 10.4)	-5.3 (-29.8, 27.8)	-4.6 (-29.4, 29.0)	-10.7 (-35.5, 23.6)	-9.2 (-34.5, 25.9)	-23.1 (-35.7, -8.1)	-20.6 (-33.6, -5.1)	-26.7 (-40.3, -10.0)	-23.7 (-38.0, -6.4)	-18.9 (-32.4, -2.7)	-19.0 (-32.6, -2.9)
L-BMI/H-TyG	-0.8 (-10.7, 10.2)	-0.4 (-11.0, 11.4)	-4.5 (-22.7, 17.9)	-8.4 (-26.9, 14.7)	-5.2 (-24.6, 19.3)	-8.7 (-28.5, 16.6)	-24.6 (-33.5, -14.4)	-21.7 (-31.5, -10.4)	-28.4 (-38.1, -17.2)	-24.8 (-35.4, -12.2)	-20.2 (-29.7, -9.2)	-21.2 (-31.3, -9.6)
M-BMI/H-TyG	-7.9 (-15.8, 0.8)	-7.4 (-16.5, 2.6)	-4.9 (-20.6, 13.9)	-9.8 (-26.6, 11.0)	-12.3 (-27.9, 6.7)	-16.4 (-33.2, 4.6)	-26.2 (-33.8, -17.8)	-23.3 (-32.2, -13.3)	-29.7 (-38.0, -20.5)	-26.0 (-35.7, -14.8)	-24.8 (-32.6, -16.1)	-25.9 (-34.8, -16.1)
H-BMI/H-TyG	-3.4 (-13.3, 7.6)	-2.8 (-13.8, 9.7)	-7.7 (-25.6, 14.7)	-11.6 (-30.7, 12.9)	-10.7 (-29.4, 13.0)	-13.9 (-33.9, 12.1)	-29.6 (-38.1, -19.9)	-25.6 (-35.7, -14.1)	-33.0 (-42.3, -22.3)	-28.0 (-39.0, -15.0)	-26.7 (-35.7, -16.4)	-28.1 (-38.0, -16.7)

Estimated percent changes derived from linear regression models with natural log-transformed semen parameters. Model 1 was adjusted for age. Model 2 was adjusted for age, education level, abstinence duration, smoking status and dyslipidemia. Ref, reference group; BMI, body mass index; TyG, triglyceride-glucose index L-BMI, low-BMI; M-BMI, middle-BMI; H-BMI, high-BMI; L-TyG. low-Ty; H-TyG, high-TyG.

**Figure 1:**
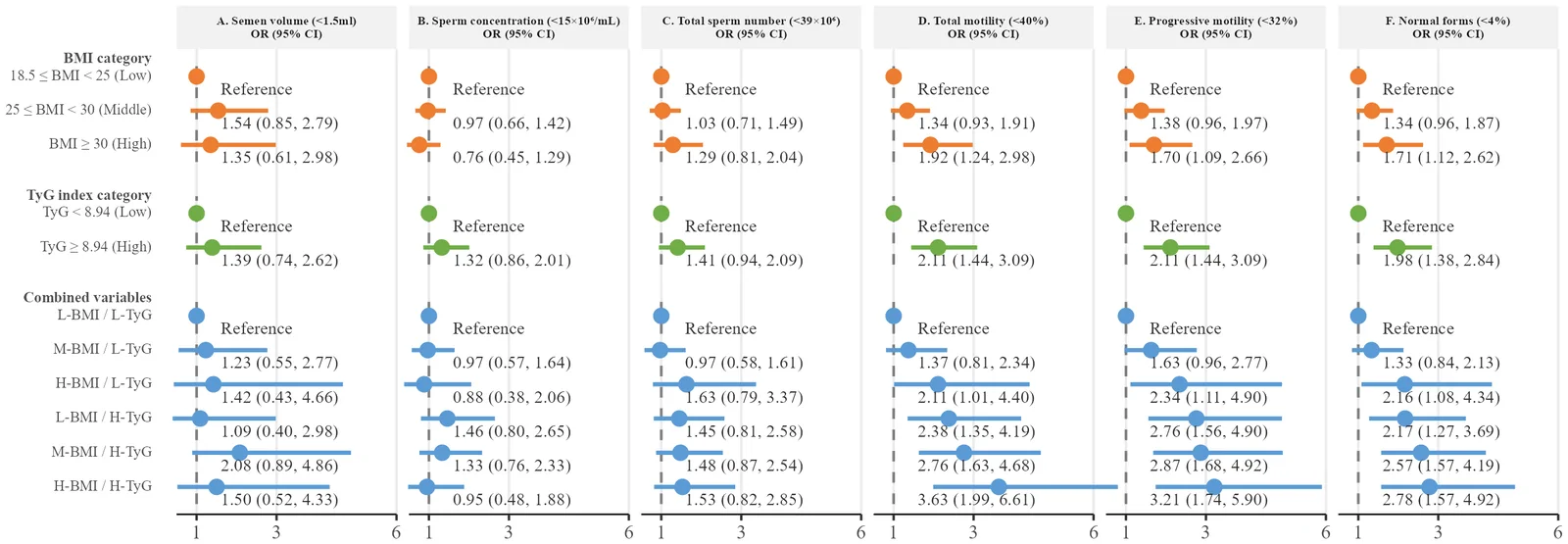
Odds ratios and 95% confidence intervals for reduced semen quality parameters according to BMI category, TyG index category, and combined BMI and TyG phenotypes: **(A)** semen volume <1.5 mL; **(B)** sperm concentration <15 × 10^6^/mL; **(C)** total sperm number <39 × 10^6^; **(D)** total motility <40%; **(E)** progressive motility <32%; and **(F)** normal forms <4%. Models were adjusted for age, education level, abstinence duration, smoking status, and dyslipidemia.

Analyses of combined BMI–TyG phenotypes showed a clear gradient of risk across BMI-TyG categories (**Figure 1**). Men in the H-BMI/H-TyG group exhibited the greatest reductions in semen quality and the highest odds of decline, reaching ORs of 3.63 (95% CI:1.99, 6.61) for total motility, 3.21 (95% CI:1.74, 5.90) for progressive motility, and 2.78 (95% CI:1.57, 4.92) for normal forms after full adjustment. The L-BMI/H-TyG group showed numerically higher impairment rates than the H-BMI/L-TyG group across sperm motility and morphology outcomes. No significant associations were observed for semen volume, sperm concentration, or total sperm number across any individual or combined groups.

### Interaction analyses of BMI and TyG index categories with semen quality

No statistically significant multiplicative interaction between BMI and TyG index categories was observed for any semen quality parameter (all P for interaction > 0.05; **Supplementary Table 2**).

### Predictive value of BMI and TyG index for semen quality

ROC analyses showed that BMI and TyG provided limited discrimination for semen volume, sperm concentration, and total sperm number (AUCs < 0.650) (**Figure 2**). In contrast, the combined BMI-TyG model achieved the highest AUCs for total motility (0.720, 95% CI: 0.684, 0.757), progressive motility (0.712, 95% CI: 0.675, 0.750), and normal forms (0.658, 95% CI: 0.619, 0.697), and these AUCs were significantly higher than those of the baseline model according to DeLong tests. Compared with the BMI-based model, the addition of TyG improved discrimination and reclassification for impaired sperm motility and morphology outcomes, as reflected by positive continuous NRI and IDI estimates (NRI range:0.212 to 0.321, IDI range:0.032 to 0.036; all P < 0.01; **Supplementary Tables 3**–**8**).

**Figure 2:**
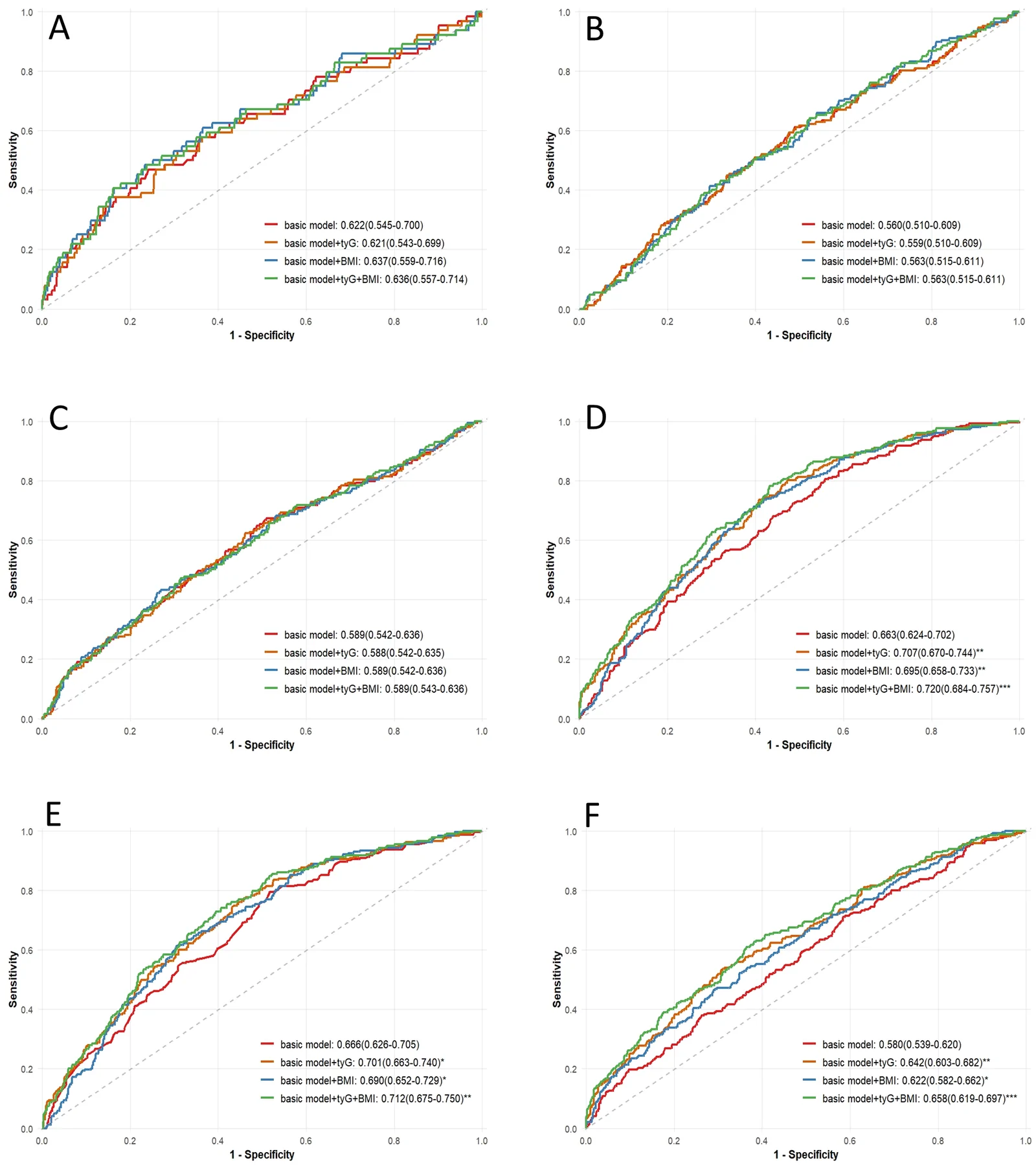
Receiver operating characteristic (ROC) curves comparing model predictive value for reduced semen quality parameters: **(A)** semen volume <1.5 mL; **(B)** sperm concentration <15×10^6^/mL; **(C)** total sperm number <39×10^6^; **(D)** total motility <40%; **(E)** progressive motility <32%; and **(F)** normal forms <4%. The base model included age, education level, abstinence duration, smoking status and dyslipidemia. The asterisks indicate the level of statistical significance for the comparison against the basic model using DeLong's test: *P<0.05, **P < 0.01, ***P<0.001.

### Exploratory mediation analysis of TyG index in the association between BMI and semen quality

Exploratory mediation analysis was performed to quantify the extent to which TyG statistically accounted for the observed association between BMI and semen parameters (**Figure 3**). Significant indirect effects were observed for total motility, progressive motility, and normal forms. The TyG index statistically accounted for 35.9%, 33.9%, and 28.5% of the observed association between BMI and these parameters, respectively (all P < 0.001). In contrast, no significant indirect effects were identified for semen volume, sperm concentration, or total sperm number.

**Figure 3:**
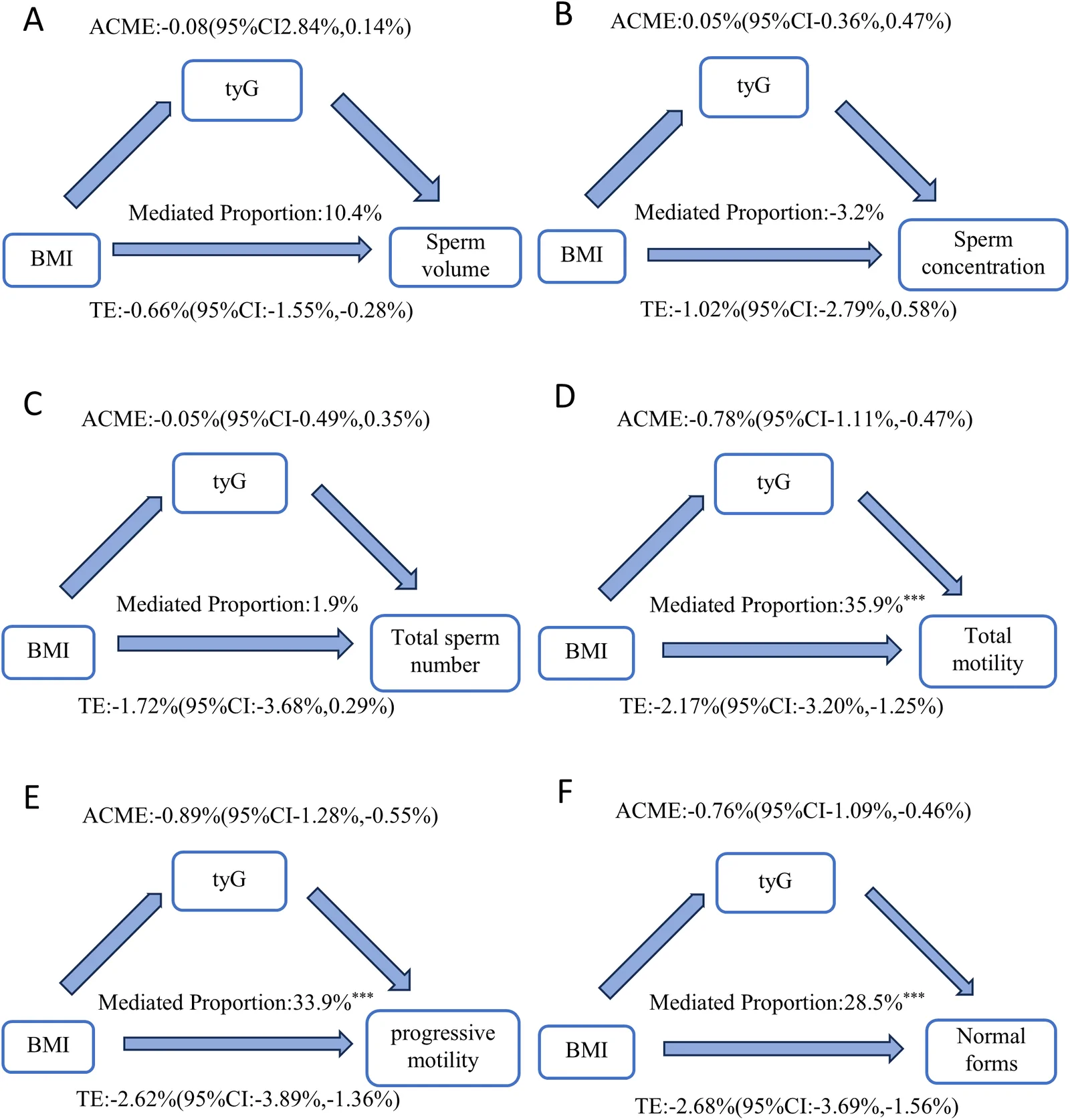
Exploratory mediation analyses of the TyG index in the associations between BMI and semen quality parameters: **(A)** semen volume; **(B)** sperm concentration; **(C)** total sperm number; **(D)** total motility; **(E)** progressive motility; and **(F)** normal forms. Models were adjusted for age, education level, abstinence duration, smoking status and dyslipidemia. ACME, average causal mediation effect; TE, total effect. ***P < 0.001.

### Subgroup analysis

Subgroup analyses stratified by age (<35 and ≥35 years) showed generally consistent associations between combined BMI/TyG categories and semen quality parameters. Associations of combined BMI and TyG categories with lower total motility, progressive motility, and normal forms were broadly consistent across both age groups. Although slightly larger reductions in normal forms were observed among younger men, no significant interaction by age was detected for any semen parameter (all P for interaction > 0.05), indicating that the observed associations were broadly comparable across age strata.

## Discussion

This retrospective cross-sectional study showed that elevated BMI and TyG were independently associated with impaired sperm motility and normal morphology. Combined BMI–TyG phenotypes further differentiated semen quality impairment, with the H-BMI/H-TyG group showing the highest risk and the L-BMI/H-TyG group showing numerically higher impairment rates than the H-BMI/L-TyG group across sperm motility and morphology outcomes. These findings suggest that BMI alone may not fully capture metabolic risk related to semen quality and that TyG may provide complementary information for metabolic risk stratification.

Our findings agree with previous clinical studies linking obesity to impaired sperm motility and morphology (22, 23). Excess adiposity appears to harm sperm function through multiple converging pathways. These include disruption of hypothalamic-pituitary-gonadal axis signaling (24), promotion of chronic low-grade inflammation (25), and increased scrotal heat accumulation (26). Not all obese men, however, exhibit impaired semen quality. Some remain normal despite excess adiposity (10, 17). This inconsistency likely reflects BMI’s inability to capture metabolic dysfunction beyond adiposity. Complementary metabolic indicators may therefore be useful in future studies of male reproductive health.

IR is a core component of metabolic dysfunction. It often coexists with obesity, with the two influencing each other (27). The TyG functions as a well-validated indicator of IR that performs comparably to HOMA-IR while requiring only routinely measured biochemical parameters (28, 29). In male reproductive medicine, Belladelli et al. (30) noted that elevated TyG was correlated with higher odds of oligozoospermia (OR = 1.58) in a cohort of 726 Italian men. Extending this limited evidence, we observed that TyG was related to reductions in semen motility, progressive motility, and normal forms. These results align with previous reports linking IR to impaired semen quality parameters (13, 31).

No prior study has examined the combined association of BMI and TyG index with male semen quality. Consistent with the notion of metabolically obese normal-weight individuals (32), we observed a clear gradient of semen quality impairment across combined BMI–TyG phenotypes. Relative to the L-BMI/L-TyG reference, men in the H-BMI/H-TyG group had 3.63-fold, 3.21-fold, and 2.78-fold higher odds of impaired sperm motility, reduced progressive motility, and abnormal morphology, respectively. Although no statistically significant multiplicative interaction between BMI and TyG was observed, the combination of BMI and TyG provided meaningful risk stratification. The combined model consistently achieved the highest AUCs, with improvements in discrimination and reclassification compared with either indicator alone in this study. These findings suggest that adiposity and metabolic dysfunction may provide complementary information for identifying semen quality impairment. Although no directly comparable studies exist, Bachelot et al. (33) reported that a machine learning score combining anthropometric, metabolic, and oxidative parameters was significantly associated with sperm DNA fragmentation. This provides indirect support for the value of multidimensional metabolic assessment in male reproductive health. The discrimination and reclassification findings were largely confined to sperm motility and morphology, not sperm counts. IR-related metabolic abnormalities may thus be more closely associated with sperm function than with sperm production. One possible explanation is that IR may preferentially impair mitochondrial energy metabolism and redox homeostasis. These processes are more directly related to sperm motility and morphology than sperm production itself (34, 35).

A particularly important finding was that men with normal BMI but high TyG showed numerically higher impairment rates than obese men with low TyG, a subgroup that may be overlooked when metabolic risk is assessed using BMI alone. The NRI for adding TyG to BMI ranged from 0.212 to 0.321 for sperm motility and morphology outcomes. These findings imply that relying solely on BMI may incompletely capture metabolic risk related to semen quality and TyG may help refine metabolic risk assessment alongside BMI. This interpretation is supported by growing evidence that male reproductive health is closely linked to overall metabolic status (36). Dietary and lifestyle factors should also be considered when interpreting these findings. Overall dietary quality may influence semen parameters through its effects on adiposity, glycemic status, lipid metabolism, and oxidative stress, and may therefore confound or modify the associations of BMI and TyG with semen quality (37). TyG is routinely available in clinical practice. It may serve as a practical marker for future studies evaluating metabolic risk in male reproductive health. Its potential role in fertility evaluation warrants further validation in independent populations.

Beyond its association with semen quality, TyG may also provide additional insight into how obesity contributes to impaired semen quality. In exploratory mediation analyses, TyG statistically accounted for 35.9% of the observed association between BMI and total motility, 33.9% of that with progressive motility and 28.5% of that with normal forms. These findings suggest that IR-related metabolic dysfunction may partly explain the observed association between higher BMI and impaired semen quality, while additional obesity-associated mechanisms likely also contribute to reproductive dysfunction. Several biological mechanisms may underlie these observations. Obesity promotes chronic inflammation and ectopic lipid accumulation, contributing to systemic IR (38, 39). Impaired insulin signaling may in turn disrupt Leydig cell steroidogenesis and Sertoli cell metabolic support. This would compromise sperm motility and morphology (40, 41). Whether this pathway is amenable to intervention remains to be established.

By jointly evaluating BMI and TyG and examining discordant BMI-TyG phenotypes, the present study provides additional evidence regarding the interplay between adiposity, IR-related metabolic dysfunction, and semen quality. Mediation analyses offer preliminary insight into the extent to which TyG may contribute to the association between obesity and impaired semen quality. Several limitations should be noted. First, the cross-sectional design prevents causal inference and makes it difficult to establish the time sequence between BMI, TyG, and semen quality. The mediation analysis reflects statistical decomposition rather than true causal mediation. Second, semen parameters were assessed using a single semen sample from each participant. Given the intra-individual variability of semen quality, this may have introduced random measurement error and potential misclassification, particularly for dichotomized outcomes based on reference limits. Third, the TyG threshold was based on the study specific median rather than a clinical threshold. Although this approach allowed construction of interpretable combined BMI–TyG phenotypes, different TyG classifications may yield different risk strata. Additional unmeasured factors, including reproductive hormone, dietary composition and physical activity levels, may confound the observed associations. Finally, the sample was restricted to men from a fertility clinic; the conclusions may not extend to the general population.

## Conclusion

This study showed that elevated BMI and TyG index were independently associated with impaired sperm motility and normal morphology. The combination of BMI and TyG provided additional risk stratification, suggesting that elevated TyG may help identify metabolically at-risk men who are not fully captured by BMI alone. Longitudinal studies are needed to clarify temporal relationships and to determine whether improvement in metabolic status is associated with better semen quality.

## Data Availability

The raw data supporting the conclusions of this article will be made available by the authors, without undue reservation.
