# Supplementary material for: Combined assessment of body mass index and triglyceride-glucose index reveals metabolic risk phenotypes associated with semen quality impairment
Source: Front Endocrinol (Lausanne). 2026 Sep 24;17:1960020. doi: 10.3389/fendo.2026.1960020 (PMC13647161; doi:10.3389/fendo.2026.1960020)
Supplement: Supplementary file 1 [file DataSheet1.docx]

**Supplementary Material**

**Table S1. Distribution of semen quality parameters among the study subjects (n=804)**

| **Semen parameter** | **Mean** | **SD** | **P5** | **P25** | **Median** | **P75** | **P95** | **IQR** |
| --- | --- | --- | --- | --- | --- | --- | --- | --- |
| Semen volume (mL) | 2.7 | 1.1 | 1.1 | 2.0 | 2.5 | 3.2 | 5.2 | 1.2 |
| Sperm concentration (10⁶/mL) | 38.2 | 32.2 | 4.9 | 16.1 | 29.5 | 49.6 | 101.0 | 33.5 |
| Total sperm number (10⁶) | 100.4 | 90.8 | 10.1 | 39.3 | 72.1 | 132.7 | 278.8 | 93.4 |
| Total motility (%) | 46.5 | 18.6 | 13.0 | 34.0 | 46.0 | 60.0 | 76.0 | 26.0 |
| Progressive motility (%) | 41.8 | 18.3 | 10.0 | 29.0 | 41.0 | 55.0 | 71.8 | 26.0 |
| Normal forms (%) | 4.2 | 2.0 | 1.0 | 3.0 | 4.0 | 5.0 | 8.0 | 2.0 |

*SD, standard deviation; P5, 5th percentile; P25, 25th percentile; P75, 75th percentile; P95, 95th percentile; IQR, interquartile range (P75-P25).*

**Table S2. Estimated percent changes and 95% CIs for the multiplicative interaction between** **BMI and TyG index categories in relation to semen quality parameters**

|  | **Semen volume (mL)** | **Sperm concentration (10⁶/mL)** | **Total sperm number (10⁶)** | **Total motility (%)** | **Progressive motility (%)** | **Normal forms (%)** |
| --- | --- | --- | --- | --- | --- | --- |
| Multiplicative effect | 0.9 (-7.7, 10.4) | 4.4 (-12.9, 25.2) | 5.4 (-13.4, 28.2) | 3.9 (-6.5, 15.5) | 11.7 (-1.2, 26.3) | 5.5 (-5.4, 17.7) |
| P for interaction | 0.841 | 0.642 | 0.603 | 0.478 | 0.079 | 0.337 |
| *Models were adjusted for age, education level, abstinence duration, smoking status, and dyslipidemia.* | | | | | | |

**Table S3. NRI and IDI for predicting semen volume decline**

| **Model** | **NRI (95% CI)** | **P value** | **IDI (95% CI)** | **P value** |
| --- | --- | --- | --- | --- |
| Basic model | Reference | — | Reference | — |
| Basic + TyG | 0.153 (-0.101, 0.408) | 0.238 | 0.002 (-0.001, 0.005) | 0.144 |
| Basic + BMI | 0.200 (-0.054, 0.455) | 0.123 | 0.006 (0.000, 0.012) | 0.088 |
| Basic + TyG + BMI | 0.240 (-0.014, 0.494) | 0.065 | 0.007 (0.000, 0.013) | 0.048 |
| +TyG vs. +TyG+BMI | 0.245 (-0.009, 0.499) | 0.059 | 0.005 (-0.002, 0.011) | 0.138 |
| +BMI vs. +TyG+BMI | 0.175 (-0.077, 0.428) | 0.174 | 0.000 (-0.001, 0.003) | 0.261 |

*Reduced semen volume was defined as <1.5 mL. NRI, net reclassification improvement; IDI, integrated discrimination improvement; BMI, body mass index; TyG, triglyceride-glucose index.*

**Table S4. NRI and IDI for predicting reduced sperm concentration**

| **Model** | **NRI (95% CI)** | **P value** | **IDI (95% CI)** | **P value** |
| --- | --- | --- | --- | --- |
| Basic model | Reference | — | Reference | — |
| Basic + TyG | -0.004 (-0.175, 0.166) | 0.959 | 0.000 (0.000, 0.000) | 0.997 |
| Basic + BMI | 0.049 (-0.122, 0.219) | 0.575 | 0.000 (-0.001, 0.002) | 0.780 |
| Basic + TyG + BMI | -0.032 (-0.202, 0.138) | 0.714 | 0.000 (-0.001, 0.002) | 0.777 |
| +TyG vs. +TyG+BMI | -0.007 (-0.178, 0.163) | 0.932 | 0.000 (-0.001, 0.002) | 0.778 |
| +BMI vs. +TyG+BMI | -0.002 (-0.173, 0.168) | 0.977 | 0.000 (0.000, 0.000) | 0.970 |

*Reduced sperm concentration was defined as <15 × 10⁶/mL. NRI, net reclassification improvement; IDI, integrated discrimination improvement; BMI, body mass index; TyG, triglyceride-glucose index.*

**Table S5. NRI and IDI for predicting reduced total sperm number**

| **Model** | **NRI (95% CI)** | **P value** | **IDI (95% CI)** | **P value** |
| --- | --- | --- | --- | --- |
| Basic model | Reference | — | Reference | — |
| Basic + TyG | 0.124 (-0.036, 0.283) | 0.129 | 0.000 (-0.001, 0.001) | 0.784 |
| Basic + BMI | 0.033 (-0.127, 0.193) | 0.689 | 0.000 (-0.001, 0.002) | 0.483 |
| Basic + TyG + BMI | 0.036 (-0.124, 0.196) | 0.657 | 0.001 (-0.001, 0.003) | 0.402 |
| +TyG vs. +TyG+BMI | 0.016 (-0.144, 0.175) | 0.848 | 0.000 (-0.001, 0.003) | 0.430 |
| +BMI vs. +TyG+BMI | 0.066 (-0.094, 0.226) | 0.417 | 0.000 (-0.001, 0.002) | 0.643 |

*Reduced total sperm number was defined as <39 × 10⁶ per ejaculate. NRI, net reclassification improvement; IDI, integrated discrimination improvement; BMI, body mass index; TyG, triglyceride-glucose index.*

**Table S6. NRI and IDI for predicting reduced total motility**

| **Model** | **NRI (95% CI)** | **P value** | **IDI (95% CI)** | **P value** |
| --- | --- | --- | --- | --- |
| Basic model | Reference | — | Reference | — |
| Basic + TyG | 0.268 (0.121, 0.415) | <0.001 | 0.049 (0.032, 0.066) | <0.001 |
| Basic + BMI | 0.373 (0.227, 0.518) | <0.001 | 0.027 (0.015, 0.039) | <0.001 |
| Basic + TyG + BMI | 0.334 (0.188, 0.480) | <0.001 | 0.063 (0.044, 0.082) | <0.001 |
| +TyG vs. +TyG+BMI | 0.243 (0.095, 0.390) | 0.001 | 0.014 (0.005, 0.023) | 0.002 |
| +BMI vs. +TyG+BMI | 0.217 (0.070, 0.364) | 0.004 | 0.036 (0.022, 0.051) | <0.001 |

*Reduced total motility was defined as <40%. NRI, net reclassification improvement; IDI, integrated discrimination improvement; BMI, body mass index; TyG, triglyceride-glucose index.*

**Table S7. NRI and IDI for predicting reduced progressive motility**

| **Model** | **NRI (95% CI)** | **P value** | **IDI (95% CI)** | **P value** |
| --- | --- | --- | --- | --- |
| Basic model | Reference | — | Reference | — |
| Basic + TyG | 0.290 (0.141, 0.439) | <0.001 | 0.042 (0.027, 0.058) | <0.001 |
| Basic + BMI | 0.328 (0.179, 0.477) | <0.001 | 0.021 (0.010, 0.032) | <0.001 |
| Basic + TyG + BMI | 0.353 (0.205, 0.501) | <0.001 | 0.053 (0.036, 0.071) | <0.001 |
| +TyG vs. +TyG+BMI | 0.214 (0.064, 0.363) | 0.005 | 0.011 (0.003, 0.019) | 0.009 |
| +BMI vs. +TyG+BMI | 0.212 (0.062, 0.361) | 0.006 | 0.032 (0.019, 0.046) | <0.001 |

*Reduced progressive motility was defined as <32%. NRI, net reclassification improvement; IDI, integrated discrimination improvement; BMI, body mass index; TyG, triglyceride-glucose index.*

**Table S8. NRI and IDI for** **predicting reduced normal forms**

| **Model** | **NRI (95% CI)** | **P value** | **IDI (95% CI)** | **P value** |
| --- | --- | --- | --- | --- |
| Basic model | Reference | — | Reference | — |
| Basic + TyG | 0.372 (0.231, 0.513) | <0.001 | 0.046 (0.031, 0.062) | <0.001 |
| Basic + BMI | 0.262 (0.120, 0.405) | <0.001 | 0.028 (0.016, 0.040) | <0.001 |
| Basic + TyG + BMI | 0.371 (0.230, 0.512) | <0.001 | 0.061 (0.044, 0.079) | <0.001 |
| +TyG vs. +TyG+BMI | 0.214 (0.071, 0.357) | 0.003 | 0.015 (0.006, 0.024) | 0.001 |
| +BMI vs. +TyG+BMI | 0.321 (0.180, 0.463) | <0.001 | 0.033 (0.023, 0.047) | <0.001 |

*Reduced normal forms were defined as <4%. NRI, net reclassification improvement; IDI, integrated discrimination improvement; BMI, body mass index; TyG, triglyceride-glucose index.*

**Figure S1. Age-stratified associations of** **combined BMI–TyG phenotypes with semen quality parameters.**

Forest plots show adjusted percentage differences and 95% confidence intervals for semen quality parameters across combined BMI–TyG phenotypes, stratified by age (<35 and ≥35 years). Models were adjusted for age, education level, abstinence duration, smoking status and dyslipidemia. *P* for interaction represents the interaction between age group and combined BMI–TyG phenotype.
